# A structural perspective of human RNA polymerase III

**DOI:** 10.1080/15476286.2021.2022293

**Published:** 2022-02-08

**Authors:** Qianmin Wang, Ming Lei, Jian Wu

**Affiliations:** a Ninth People’s Hospital, Shanghai Jiao Tong University School of Medicine, Shanghai, China; b Shanghai Institute of Precision Medicine, Shanghai, China; c State Key Laboratory of Oncogenes and Related Genes, Shanghai Jiao Tong University School of Medicine, Shanghai, China

**Keywords:** RNA polymerase III, transcription, structure, disease

## Abstract

RNA polymerase III (Pol III) is a large multisubunit complex conserved in all eukaryotes that plays an essential role in producing a variety of short non-coding RNAs, such as tRNA, 5S rRNA and U6 snRNA transcripts. Pol III comprises of 17 subunits in both yeast and human with a 10-subunit core and seven peripheral subunits. Because of its size and complexity, Pol III has posed a formidable challenge to structural biologists. The first atomic cryogenic electron microscopy structure of yeast Pol III leading to the canonical view was reported in 2015. Within the last few years, the optimization of endogenous extract and purification procedure and the technical and methodological advances in cryogenic electron microscopy, together allow us to have a first look at the unprecedented details of human Pol III organization. Here, we look back on the structural studies of human Pol III and discuss them in the light of our current understanding of its role in eukaryotic transcription.

## Introduction

Different from the prokaryotes that contains only single RNA polymerase for producing all the RNA, the eukaryotic nucleus possesses of three different kinds of RNA polymerases–RNA polymerase I, II and III (Pol I, Pol II and Pol III). Pol I and Pol II produce ribosomal RNAs (rRNAs) and messenger RNAs (mRNAs) for ribosome constitution and protein synthesis, respectively. Pol III is specialized for transcribing 5S rRNA, transfer RNAs (tRNAs), and U6 spliceosomal RNA (U6 snRNA), who function mainly in translation and related biological processes. In addition, Pol III is also defined to produce other small non-coding RNA transcripts including 7SL RNA, 7SK RNA, RNase P RNA, RNase MRP RNA, huge numbers of short interspersed nuclear element (SINE)-encoded RNAs, which contribute to endoplasmic reticulum-mediated protein synthesis, Pol II activity regulation, tRNA maturation, 35S rRNA processing and pre-mRNA splicing, etc [1,2]. In growing cells, Pol III-synthesized small RNA transcripts account for around 15% of total cellular RNAs, and for example, the amount of tRNA is 10 ~ 12 fold molar excess relative to ribosomes for efficient translation [3,4]. These small transcripts are generally less responsive to regulation than Pol II-transcribed short-lived mRNAs, but their expression is a high energy-consuming process and essential for cell growth and division [3,5]. Dysregulation of Pol III-transcribed RNAs linked to defects in the Pol III transcription apparatus or to Pol III products imbalance is associated with a large variety of human diseases such as neurodegenerative disorders and various cancers [6–8].

The process of transcription involves three main stages–initiation, elongation and termination, in which initiation and associated regulatory processes machines are the most complicated. Pol III initiation starts with transcription factors (TF) binding to promoters, and ends with TFIIIB being recruited to the complex and assembling Pol III. TFIIIB consists of three key subunits–TATA binding protein (TBP), B-double-prime 1 (BDP1) and a TFIIB-related factor (BRF1 or BRF2), whose overall architecture bears similarities to that of TFIIB in Pol II. The Pol III promoters are much more diverse in structure than the Pol I promoters, but not as various as the Pol II promoters [9]. Promoter elements of Pol III locates upstream and downstream of the transcription start site. Three typical types of promoters could be classified in Pol III initiation. In type I, the initial binding of TFIIIA at internal control region (ICR) recruits TFIIIC complex and subsequently recruits TFIIIB, mediating the assembly of Pol III to begin the transcription of 5S rRNA genes. Type II promoters, consisting of internal A- and B-box, recruits the same factors with type I except that in this case, the promoter elements recruit TFIIIC directly without participation of TFIIIA, in which tRNA and SINE genes could be specifically transcribed. Type III promoters, containing upstream TATA-box and proximal sequence element (PSE), are recognized by the multisubunit snRNA activating protein complex (SNAPc) and TFIIIB, and could direct producing U6 snRNA, RNase P RNA and 7SK RNA, etc [9]. Moreover, there is a group of hybrid promoters containing the sequence elements from type II and type III, present in selenocysteine tRNA gene (tRNA^Sec^), 7SL RNA gene and the Epstein Barr virus EBER gene [6].

All multisubunit RNA polymerases are derived from a common ancestor, a fact which becomes apparent from their amino acid sequences, subunit composition, structures and functions. Compared to Pol I and Pol II, Pol III is the most complicated RNA polymerase which contains 17 subunits in both yeast and human [2,10]. Pol III comprises a conserved core region with 10 protein components and several peripheral subcomplexes–the RPC8-RPC9 stalk, the RPC3-RPC6-RPC7 heterotrimer and the RPC4-RPC5 heterodimer (Table 1). The RPC3-RPC6-RPC7 heterotrimer has been reported to be required for transcription initiation and functions as TFIIE related in Pol II [11,12]. The heterodimer RPC4-RPC5 is involved in both transcription initiation and termination [13,14]. Despite the similarity of these complexes, the organisms that depend on them are diverse, ranging from microorganisms to human. In this review, we introduce the most recent discoveries regarding the human Pol III structural features and discuss the important advances. We then briefly discuss Pol III negative regulator MAF1 and Pol III dysfunction associated diseases. Finally, we indicate structures that are still lacking and represent important missing puzzle pieces sought to obtain a more comprehensive understanding of Pol III transcription.

**Table 1.** Subunits of archaea, yeast and human RNA polymerases

	Archaea	Eukarya					
	RNAP	Pol I		Pol II		Pol III	
		Yeast	Human	Yeast	Human	Yeast	Human
**Polymerase Core**	Rpo1	A190	RPA1	Rpb1	RPB1	C160	RPC1
	Rpo2	A135	RPA2	Rpb2	RPB2	C128	RPC2
	Rpo3	AC40	RPAC1	Rpb3	RPB3	AC40	RPAC1
	Rpo11	AC19	RPAC2	Rpb11	RPB11	AC19	RPAC2
	Rpo5	Rpb5	RPABC1	Rpb5	RPABC1	Rpb5	RPABC1
	Rpo6	Rpb6	RPABC2	Rpb6	RPABC2	Rpb6	RPABC2
	Rpo8	Rpb8	RPABC3	Rpb8	RPABC3	Rpb8	RPABC3
	Rpo10	Rpb10	RPABC4	Rpb10	RPABC4	Rpb10	RPABC4
	Rpo12	Rpb12	RPABC5	Rpb12	RPABC5	Rpb12	RPABC5
	Rpo13						
		A12.2 N-Zn-ribbon	RPA12 N-Zn-ribbon	Rpb9	RPB9	C11 N-Zn-ribbon	RPC10 N-Zn-ribbon
**TFIIS** **C-Zn-ribbon like**		A12.2 C-Zn-ribbon	RPA12 C-Zn-ribbon	TFIIS C-Zn-ribbon	TFIIS C-Zn-ribbon	C11 C-Zn-ribbon	RPC10 C-Zn-ribbon
**Polymerase Stalk**	Rpo4	A14		Rpb4	RPB4	C17	RPC9
	Rpo7	A43	RPA43	Rpb7	RPB7	C25	RPC8
**TFIIF-related**		A49	RPA49	Tfg1	TFIIFα	C53	RPC4
		A34.5	RPA34	Tfg2	TFIIFβ	C37	RPC5
**TFIIE-related**	TFEα			Tfa1	TFIIEα	C82	RPC3
	TFEβ			Tfa2	TFIIEβ	C34	RRC6
						C31	RRC7

## A brief history of human RNA polymerase III

In comparison with the yeast Pol III, human Pol III composition was identified and characterized to fall behind. The first cloned and identified human Pol III subunit is RPC4 by screening the temperature-sensitive NB51 cell-cycle mutant of baby hamster kidney cells in 1993 [15]. Three years later, the Roeder laboratory extracted and identified 15 subunits of Pol III from Hela cell nuclear extraction using several steps of conventional chromatography, and characterized the cooperation between TFIIIC1 and TFIIIC2 when presented simultaneously on the promoters of VA1 or tRNA1 (also as tRNA^Met^) templates [16]. Soon later, the Roeder laboratory further optimized the human Pol III extract procedures by establishing the Flag-tagged RPC4 as affinity purification bait in Hela cell line, and then characterized the heterotrimer RPC3-RPC6-RPC7 as a transcription initiation specific subcomplex, which is loosely associated with the human Pol III core region [17]. Until then the available information of several subunits, such as RPC2 and RPC5, remained ambiguous to their molecular weight on SDS-PAGE analysis and the limited sequence information. The Hernandez laboratory further contributed to entirely characterize the composition of human Pol III, and reconstitution of transcription initiation complex containing SNAPc, TFIIIB and Pol III with transcription activity on the human U6 snRNA promoter [18,19].

Owing to the large size and the multi-subunit composition, it remained very challenging to gain the whole complex scene of human Pol III for many years. Nevertheless, several human Pol III sub-complexes and Pol III-related transcription initiation complexes were achieved by X-ray crystallography. The structure of human RPC3 was solved in 2011, which revealed that RPC3 folds into four tandem extended winged-helix (WH) domains (referred to as WH1-4) and WH3 is required for the interaction with RPC6 [20]. Later, the structure of RPC3 in complex with the core region of RPC7 was determined, in which RPC7 was defined as a bridge in between WH1, WH2 and the coiled coil domain of RPC3 [21]. The first atomic insight into the human Pol III transcription preinitiation complex (PIC) came from structural studies of the TFIIIB sub-complex with double-stranded DNA (dsDNA), in which the essential SANT domain of BDP1, together with TBP and BRF2 embraced tightly the 25-bp dsDNA fragment of human U6 snRNA promoter [22]. Recently, improvement of endogenous extract and purification procedure and development of cryogenic electron microscopy (cryo-EM) technology have accelerated our pace on understanding human Pol III, and also have led to a first glimpse on high-resolution structures of human Pol III [23–26].

## The conserved catalytic core of human Pol III

### The active centre

**Figure 1.**
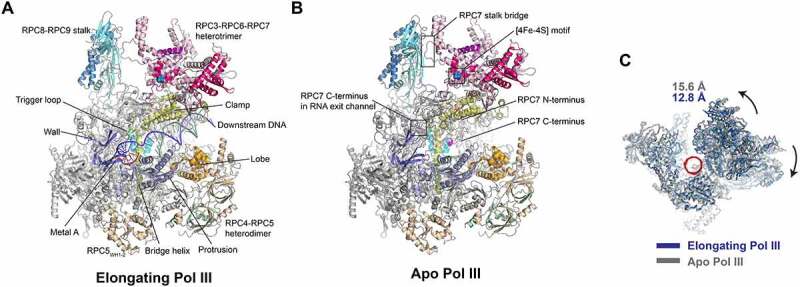
**Human RNA polymerase III (Pol III) structure and function**. (A) Important structural elements of the elongating Pol III including the bridge helix; the clamp; the trigger loop; the wall; the protrusion; the lobe; the peripheral RPC8-RPC9 stalk; the RPC3-RPC6-RPC7 heterotrimer and the RPC4-RPC5 heterodimer [Protein Data Bank (PDB) ID 7D58, 7AE1 and 7DU2]. (B) Important structural elements of the apo Pol III including the [4Fe-4S] motif in RPC6; the RPC7 stalk bridge and the RPC7 C-terminus in the active cleft and the RNA exit channel (PDB ID 7D59 and 7A6H). (C) Front view on open and closed clamp conformations in human Pol III with the closed clamp state in blue and open clamp state in grey. The corresponding values indicate the relative distance of the cleft of the two conformations. The black arrows indicate the orientation of the movement of heterotrimer and stalk during the transition from apo state to elongating state.

**Figure 2.**
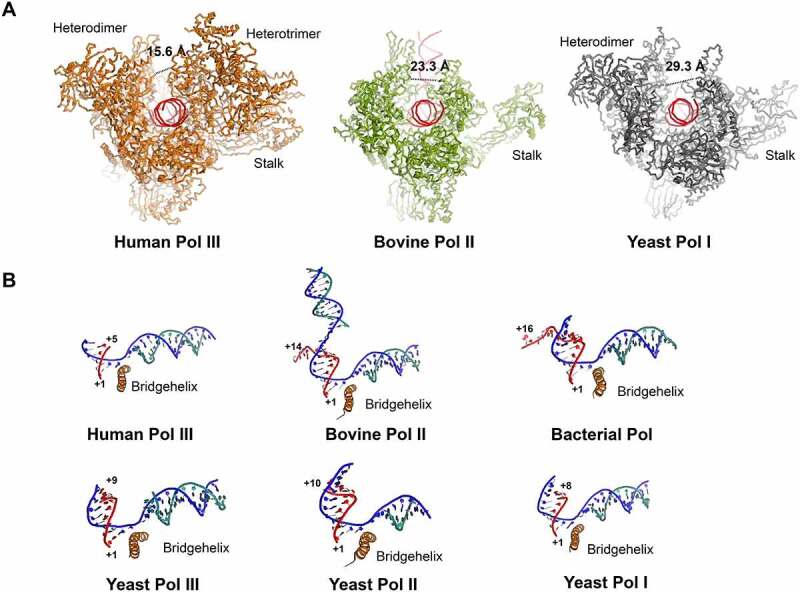
**The structural features of polymerase active centre in the elongating state**. (A) Front view of elongating Pol III (left panel, PDB ID 7D58), Pol II (middle panel, PDB 5FLM) and Pol I (right panel, PDB ID 5M3F). The cleft distance is indicated by a dished line and the corresponding values indicate the relative distance of the cleft. (B) Close-up view of the active site of human Pol III (PDB ID 7D58), bovine Pol II (PDB ID 5FLM), bacterial Pol (PDB ID 2O5I), yeast Pol III (PDB ID 5FJ8), yeast Pol II (PDB ID 1Y1W) and yeast Pol I (PDB ID 5M3F).

The general architecture of human Pol III active centre, including the downstream DNA binding cleft, the RNA exit channel, as well as the secondary channel, are highly conserved from yeast to human (Figure 1a,b) [23–27]. In addition, a universal conformation transition adopted by RNA polymerases in bacteria and yeast was also captured between human apo and elongating Pol III, with the contraction of the DNA binding cleft in the elongating state (Figure 1c) [23,25,26]. Compared with other kinds of RNA polymerases, one of the distinct features is that the DNA-binding cleft of human Pol III adopts the narrowest conformation (Figure 2a). In general, during the elongating state, the DNA-binding cleft of human Pol III is about 14 Å and 8 Å narrower than those of yeast Pol I and mammalian Pol II, respectively (Figure 2a) [28–30]. Another remarkable feature is that only 5–6 bp DNA/RNA hybrid was observed at the active centre of human elongating Pol III, whereas the previously reported other kinds of RNA polymerases could sustain longer DNA/RNA hybrid. For example, 8 bp DNA/RNA hybrid model could be clearly built in the cryo-EM structure of yeast elongating Pol I, and electron density allowed definite tracing of 9 bp DNA/RNA hybrid together with non-template DNA and upstream dsDNA in mammalian elongating Pol II (Figure 2b) [29–31]. The short and week DNA/RNA hybrid in Pol III could probably reason from the relatively looser association of DNA/RNA hybrid with the active centre [25,27]. These observations raise the possibility that the active centre of Pol III is capable of intrinsically week DNA/RNA hybrid binding, which would play a role as key determinant for Pol III termination [32]. Collectively, comparing with other kinds of RNA polymerases, Pol III has a relatively tight downstream dsDNA-binding cleft and a week DNA/RNA hybrid binding pocket, which together enable the efficient transcription reaction and termination.

### RPC10

**Figure 3.**
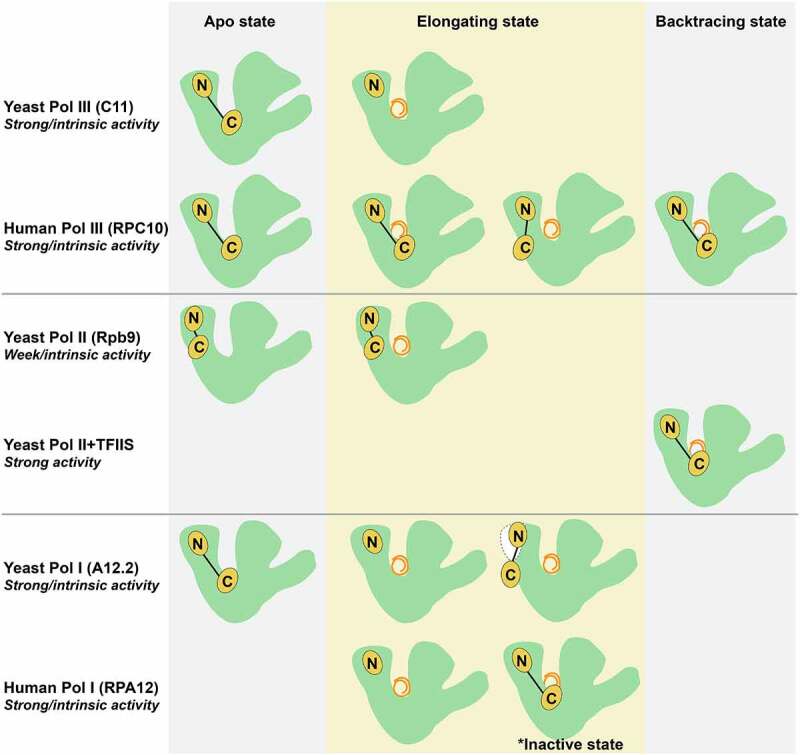
**Schematic presentation of the RPC10 C-Zn-ribbon domain and its homologs in Pol I, Pol II and Pol III during apo, elongating and backtracking states**. In the apo state, the C-Zn-ribbon of C11/RPC10/A12.2 with strong intrinsic endonuclease activity in yeast Pol III, human Pol III and yeast Pol I locate in the active site, while the C-Zn-ribbon of Rpb9 with very weak intrinsic endonuclease activity in yeast Pol II resides on the enzyme surface. In the elongating state, the RPC10 C-Zn-ribbon of human Pol III shows either ‘inside active site’ conformation, or the ‘outside funnel’ conformation, while the C11 C-Zn-ribbon of yeast Pol III disassociate from the enzyme core; the C-Zn-ribbon domain of A12.2 and RPA12 in both yeast and human Pol I are excluded from the active site, and A12.2 C-Zn-ribbon only visible on the yeast Pol I surface when A49-A34.5 dissociate from enzyme, and RPA12 C-Zn-ribbon was observed in the active site in an inactive state. In the backtracking state, the RPC10 C-Zn-ribbon of human Pol III still stays in the active site; the elongation factor TFIIS with strong endonuclease activity binds to yeast Pol II with the C-Zn-ribbon domain in the active site.

Despite having a small molecular weight (12.3 kDa), RPC10 performs multi-functions in both transcription elongation and termination. The N-terminal zinc ribbon domain (N-Zn-ribbon) of RPC10 resembles RPB9 in Pol II and the N-Zn-ribbon of A12.2 in Pol I, which locates into the cavity formed by RPC1, RPC2 and RPC4-RPC5 heterodimer. Following the N-Zn-ribbon, a long-linker region with an extended conformation meanders through the RPC1 jaw domain and the funnel helix. The C-terminal zinc ribbon domain (C-Zn-ribbon) of RPC10 is functional equivalence to the elongation factor TFIIS in Pol II and the C-Zn-ribbon of A12.2 in Pol I. In all the C-Zn-ribbon domains, there is a conserved acidic loop that is essential for endonuclease catalysis [33]. This precise RNA cleavage activity is required for RNA polymerases to prevent misincorporations during elongation as transcriptional proofreading [34,35]. Recent structural studies of human Pol III revealed that the C-Zn-ribbon could stay near the active centre in both apo and elongating state, with the acidic loop inserting into the secondary channel (Figure 3) [23,25,26]. This observation about the RPC10 C-Zn-ribbon is comparable with TFIIS in Pol II, as well as its A12.2 counterpart in apo Pol I and raises a possibility that RPC10 in human Pol III might employ consistently surveillance mechanism to ensure the transcription fidelity, and also to probably facilitate the transcription reinitiation (Figure 3) [29,30,36]. However, in the elongating state of Pol I, the C-Zn-ribbon of A12.2 is excluded from the active centre [37–39]. Notably, the Mueller group also determined that human RPC10 adopts another so-called ‘outside funnel’ conformation, with the C-Zn-ribbon folding back and positioning to the RPC1 jaw domain [26], suggesting a contribution to Pol III-dependent promoter melting.

Although RPC10 is highly conserved among different species, the yeast homolog C11 shows different molecular architecture. Structurally, the N-Zn-ribbon of C11 is in close connection with C53-C37 dimerization [27]. The C-Zn-ribbon of C11 could be only observed in the apo state of yeast Pol III at a very low-density threshold, while no according density could be reconstituted in the elongation or pre-initiation complex [27,40–42]. Therefore, the yeast and human Pol III adopt different conformation regarding RPC10/C11, suggesting the different functional roles between species. In general, functional characterization of yeast C11, together with the heterodimer C53-C37, determined that C11 participated in transcription termination [33,43–45]. The significance of C11 during the transcription termination was identified by establishing the Pol IIIΔ variant, lacking C11, C37 or C53 counterparts in *Schizosaccharomyces pombe*. The phenotype results showed C53-C37 is required for termination signal recognition, and C11 is necessary for promoting the DNA/RNA hybrid instability and RNA release [13,33,43]. A very recent study about C11 further specified that the N-Zn-ribbon and the C-Zn-ribbon of C11 play independent roles in termination and endonuclease activity, while the linker region between the N-Zn-ribbon and the C-Zn-ribbon of C11 are required for reinitiation-recycling activity of Pol III [44].

## The peripheral module of Pol III

### The stalk

The human Pol III stalk comprises RPC8 and RPC9, homologous to RPB7-RPB4 in human Pol II and A43-A14 in yeast Pol I [10,28]. RPC8 contains an oligonucleotide/oligosaccharide-binding fold (OB-fold) domain and a tip domain, while RPC9 has a compact N-terminal tip-associated domain and a helicase and RNase D C-terminal (HRDC) domain at the C-terminus. The tip domain of RPC8 forms a wedge and inserts into the pocket constituted by RPC1, RPC2 and RPABC2. Interestingly, the extremely N- and C-terminal tails of RPC1 extends from Pol III core and sandwiches tightly the stalk module. All these interactions make the stalk module adhere on the surface of Pol III core, consistent with the cryo-EM structures of yeast Pol III [27,40–42,46]. Functional analyses using mutagenesis on C25 (RPC8 homolog in yeast) and domain deletion on C17 (RPC9 homolog in yeast) revealed that the mutant Pol III were defective in the specific synthesis of pre-tRNA transcripts, but were indistinguishable from wild type in transcript elongation, cleavage and termination, reinforcing the notion that C25 and C17 are critical for transcription initiation in Pol III [46,47]. Furthermore, the electrophoresis mobility shift assay (EMSA) results showed that C17-C25 has nucleic acids binding activity with strong affinity to tRNA *in vitro*, suggesting that C17-C25 might bind the nascent transcripts emerging from the adjacent exit pore of Pol III [46].

### The RPC3-RPC6-RPC7 heterotrimer

In human Pol III, RPC3, RPC6 and RPC7, which form a stable heterotrimer subcomplex and loosely associate with Pol III core [11,17], are defined to be required specifically for transcription initiation. Topologically similar with the architecture of yeast Pol III, the RPC3-RPC6-RPC7 heterotrimer sits on top of the clamp head, adjacent to the stalk module of human Pol III (Figure 1a,b) [23–27]. Although the RPC3-RPC6-RPC7 heterotrimer in Pol III and TFIIE in Pol II are identified to be structural and functional equivalent between each other, some remarkable differences are present in their interaction mode with each stalk module, respectively. In Pol II, the zinc-ribbon domain of the large TFIIE subunit directly recognized the OB-fold domain of RPB7, connecting the interaction of TFIIE and stalk module [48,49]. Nevertheless, a loop region contains residues Tyr73-Trp99 of RPC7 extruded from RPC3-RPC6-RPC7 heterotrimer onto the RPC8-RPC9 heterodimer, bridging the association, like a tether, between the heterotrimer and the stalk module in Pol III (Figure 1a,b) [25,26]. The five-residue patch Asp83-Tyr87 of RPC7 closely interacts with the stalk module, especially through the cation-π interaction between Tyr87 of RPC7 and Arg107 of RPC8^26^. It is noteworthy that this ‘stalk bridge’ motif is highly conserved in RPC7 proteins from yeast to human. Deletion of the five-residue patch in C31 (RPC7 homolog in yeast) results in a lethal phenotype, underscoring the essential function of RPC7-mediated interaction [25]. Genetic results, together with the structural comparison analyses, revealed that the ‘stalk bridge’ of RPC7 is involved in the dynamic movement of heterotrimer and stalk module during the transition between apo and transcribing states, which is an indispensable feature of Pol III.

Previous studies showed that deletion of various residues from the conserved C-terminal acidic tail of C31 leads to severe yeast growth defect. Nevertheless, this tail region was not completely observed in all reported yeast Pol III structures, remaining the mechanical significance of this conserved acidic tail unclear [11]. Recently, the high-resolution cryo-EM structures of human Pol III clarified this enigmatic issue (Figure 1a,b). In contrast to the transcribing Pol III structure, it was unanticipated to observed that the C-terminal acidic tail of RPC7 was located in the vicinity near the double strand DNA binding cleft, DNA/RNA hybrid position, as well as the RNA exiting channel in the apo Pol III structure, indicating that non-transcribing Pol III is probably autoinhibited by the C-terminal acidic tail of RPC7, which could prevent the engagement of Pol III in non-specific transcription [25]. Interestingly, the last four amino acids of RPC7 observed in the RNA exiting channel is in close vicinity to the general transcription initiation factor BRF1 when modelled the transcription pre-initiation complex of human Pol III by docking the yeast TFIIIB complex structure onto the human apo Pol III structure, suggesting that RPC7 might be also required to cooperate with TFIIIB and facilitate the transcription initiation [25].

Similar regulating feature was also observed in the crystal structures of yeast Pol I, in which an acidic ‘expander’ or ‘DNA-mimicking loop’ of RPA1 was inserted into the catalytic cleft of yeast Pol I [29,30]. A marked difference in the function of these loops from RPA1 and RPC7 is that the acidic ‘expander’ of RPA1 in Pol I is at nonessential nature; however, the C-terminal acidic tail of RPC7 in Pol III is essential for cellular viability [11,25]. It would be interesting to understand the exact regulating basis of these acidic motifs in both Pol I and Pol III. Biochemical tests showed that the elongating Pol III could be obtained effectively from the endogenously extracted apo Pol III incubated with excess DNA/RNA scaffold, indicating that the active centre of Pol III shows higher binding affinity to DNA/RNA scaffold than to the C-terminal acidic tail of RPC7 *in vitro*. With regard to the complexity of transcription regulation in cells, future experiments are needed to dissect how the C-terminal acidic tail of RPC7 contribute to Pol III-dependent transcription initiation *in vivo*.

In contrast to yeast C31, two isoforms of mammalian, RPC7–RPC7α and RPC7β, have been identified, corresponding to two mammalian Pol III forms–Pol IIIα and Pol IIIβ, respectively [50]. Genome-wide chromatin immunoprecipitations followed by high-throughput sequencing (ChIP-seq) analyses in mouse normal liver and cultured hepatocarcinoma cells revealed that Pol IIIα and Pol IIIβ perform the same pattern of genome-wide occupancy [51]. However, a variable expression level of RPC7α and RPC7β was observed under various cultured conditions or in different type of cells. RPC7α is specifically expressed in embryonic stem cells and following tumoural transformation. The promoter of the gene encoding RPC7α, as well as other Pol III gene promoters, binds the transcription factor MYC [51]. Nevertheless, there is no detectable MYC occupancy at the promoter of gene encoding RPC7β, which displays a broad expression pattern in differentiated and undifferentiated cells [50,51]. In aggregate, the two isoforms of RPC7 have similar functions in target gene specificity, but probably response differently to the specific cellular regulation signals during development. A recent C31 methylation study could support the above phenotypes observed in human cells. The yeast arginine methyltransferase Hmt1 could methylate Arg5 and Arg9 in C31, which positively regulates tRNA transcription in the optimal growth conditions [52]. However, the methylation of C31 shows negatively transcriptional regulation when cells are maintained under the setting of stress, through the enhanced interaction with the Pol III master negative regulator Maf1^52^. Interestingly, in mammals the methyltransferase PRMT1 is able to methylate only RPC7β, but not RPC7α, most likely due to RPC7α lacking the conserved N-terminal arginines at position 4 and 8, providing evidence for how RPC7α supports tumoural growth by avoiding repression of Pol III transcription by the tumour suppressor MAF1, whereas RPC7β can be repressed by MAF1^52^.

A distinct difference in the structures of human RPC6 and *Saccharomyces cerevisiae* C34 is that an iron-sulphur cluster [4Fe-4S] exists at the C-terminus of human RPC6, coordinated by four cysteine residues from RPC6 (Figure 1a,b), whereas in *S. cerevisiae* C34, the cysteine-containing motif was lost and replaced by a long loop structurally [27]. Biochemical analyses of recombined RPC3-RPC6 sub-complex following mass spectrum revealed that the [4Fe-4S] cluster was bound to RPC6; however, the exact location of this cluster was still unknown [53]. The cryo-EM structures of human Pol III first provide atomic evidences that the [4Fe-4S] motif locates directly above the clamp and underneath the N-terminal loop of RPC7^23,^ [25,26]. The [4Fe-4S] cluster locates more than 70 Å from the activity site; therefore, it might not participate in the transcription reaction directly. Instead, the [4Fe-4S] motif might function in stabilizing the interaction between RPC3-RPC6-RPC7 heterotrimer and polymerase core [26]. Consistent with this hypothesis, it has been detected that the TFEβ (TFIIE homolog in archaea) also contains a [4Fe-4S] cluster, which is required for dimerization of TFEα and TFEβ, as well as the association with archaeal RNA Pol clamp [53]. Despite the [4Fe-4S] motif is missing in C34 of *S. cerevisiae*, the sequence and structural comparison show that the sequence elements on C82 might function in tethering heterotrimer to polymerase core region, which could compensate for the lacking of the [4Fe-4S] motif. In the future, more studies are needed to investigate whether this the iron-sulphur cluster in human Pol III response to the oxidation condition, and/or further involves in transcription regulation.

### The RPC4-PRC5 heterodimer

RPC4-RPC5 heterodimer is closely related to its counterpart TFIIF in human Pol II and A49-A34.5 in yeast Pol I [54]. RPC4 and RPC5, dimerized into two compact β-barrel domains, are anchored to the lobe of RPC2 in human Pol III and involved in both transcription initiation and termination (Figure 1a,b) [13,41–43]. The human Pol III subunits show a high degree of conservation comparing with their yeast counterparts except for RPC5, which contains a long C-terminal extended tail harbouring four WH domains (referred to as WH1-4) with the secondary structure prediction analysis. Functional assay results showed that this C-terminal extension of RPC5 is essential for the stability of intact Pol III complex *in vivo* [24]. Recent reported human Pol III structures revealed that the closely adjacent WH1 and WH2 domains together adhere to the core of human Pol III with two types of conformation. Specifically, the crystal structure of RPC5 WH1-WH2 domain has been reported and show a compact globular overall architecture, and this feature was further confirmed by small-angle X-ray scattering (SAXS) [24]. The cryo-EM structures of human Pol III revealed that WH1-WH2 of RPC5 binds to the outer surface of human Pol III on the RPC2 side, bridging the external 1 and RPAC2, and further spans to RPABC5 (Figure 1a,b) [26]. Another type of interaction conformation was captured in human elongating Pol III structures, in which the WH1-WH2 of RPC5 point towards the downstream dsDNA in the modelled pre-initiation complex [26]. The structural rearrangement of RPC5 WH1-WH2 domain could cater to the functional requirement of RPC5 during the transcription initiation. The structure of WH3-WH4 domain of human RPC5 was recently determined by X-ray crystallography and showed a compact elongated overall conformation [24]. However, reconstituted cryo-EM density could not trace the WH3-WH4 domain of RPC5 in all the human Pol III structures, suggestive of the indirect interaction of RPC5 WH3-WH4 with the core region of human Pol III [23–26].

## The Pol III repressor MAF1

In all eukaryotic cells, Pol III-dependent transcription is highly regulated by various factors in response to stress and growth signals. Among these factors, MAF1 was identified as a Pol III negative regulator sensing the nutrient limitation, DNA damage and secretory pathway defect [55,56]. ChIP assay revealed that following dephosphorylation by PP2A, MAF1 translocated into nucleus and colocalized with the promoters recognized and occupied by Pol III under repressing conditions, suggestive of some interactions between dephosphorylated MAF1 and Pol III [57,58]. On the other side, it has been reported that MAF1 could be phosphorylated by casein kinase II *in vitro* and phosphorylation of MAF1 resulted in the enhancement of Pol III transcription activity in both yeast and human [59]. *In vitro* pull-down assay with exogenously expressed Maf1 and C160 (RPC1 homolog in yeast) identified the direct interaction between dephosphorylated Maf1 and the N-terminal 235 residues of C160 in yeast, suggesting Maf1 physically block the critical region to repress Pol III transcription activity [57]. In addition, Maf1 also contacts directly with Brf1, the general transcription initiation factor, implying that Maf1 is able to prevent assembly of the transcription initiation complex [60].

The first breakthrough in the structural characterization of Pol III-Maf1 complex occurred in 2010. With a limited 18.5 Å-resolution cryo-EM density, Maf1 could be roughly traced on the top of the Pol III clamp and induce a rearrangement of C82-C34-C31 heterotrimer [61]. Ten years later, the Mueller laboratory reported the cryo-EM structure of yeast Pol III-Maf1 at 3.3 Å resolution. It revealed that Maf1 binds to the Pol III clamp and protrusion domains, indicating that Pol III transcription inhibition by Maf1 is achieved by blocking the binding interface of the general transcription initiation factor TFIIIB [62]. Interestingly, the structural comparison of yeast Pol III-Maf1 and human Pol III uncovered that MAF1 is not compatible with RPC7α binding in the apo state of human Pol III [26,62], implying that RPC7α-containing Pol III enriched in embryonic stem cells and cancer cells escapes the negative controlling from MAF1. Consistent with this hypothesis, biochemical tests in cultured HEK293 cell line failed to reassemble a stable human Pol III-MAF1 complex.

## Pol III-related human diseases

It has been reported that a plethora of recessive mutations in Pol III subunits underlie a various kind of human disorders, such as hypomyelinating leukodystrophy (HLD), Treacher Colllins syndrome (TCS), Wiedemann-Rautenstrauch syndrome (WRS) and Varicella Zoster Virus (VZV) susceptibility [6,8]. Among these disorders, the HLD is the most common and extensively identified neurodegenerative disorders. Recessive mutation in the genes encoding the two largest Pol III subunits RPC1 and RPC2 were identified to cause HLD [63–65]. Later, leukodystrophy-causing mutations were also identified in the gene encoding RPAC1, by impairing assembly and nuclear import of RPAC1 in Pol III, but not Pol I [66]. Structure mapping showed that some mutations distribute in the core of the subunit, and some locate in the bridge helix, the trigger loop or the interface between the subunits [26]. Thus, it seems that the disease-causing mutations affect not only one specifical function of Pol III, but multiple features in respect of stability, assembly and/or activity of Pol III [67]. Besides the essential transcription function in nuclear, Pol III has been also identified playing roles in immune response in cytoplasm [68,69]. Several mutations in genes encoding RPC1, RPC3, RPC5 and RPC6 have been reported to impair immune response to VZV infection, with marginal effect of Pol III-dependent gene transcription in nucleus [70–72]. Structure analyses revealed VZV infection-related mutations distribute mainly on the periphery subcomplex and the surface of Pol III, probably disrupt the cytosolic DNA-sensing activity of Pol III [24,26].

## Concluding remarks

Since Pol III was discovered more than five decades ago, our understanding on Pol III participated transcription improved considerably. The main components of Pol III are determined, the architectures of these complexes are emerging, and our knowledge of transcription regulation is increasing. However, many fundamental questions still need to be addressed before we fully comprehend the basal mechanism and beyond. What are the molecular architectures of Pol III preinitiation complex on different type of promoters? How the Pol III transcription termination and reinitiation happen? How the Pol III pass through chromatin? What are the detailed molecular mechanisms of Pol III-related human diseases?
